# Evaluation of the Use of Project-Based Learning in the Nursing Degree

**DOI:** 10.3390/nursrep13040136

**Published:** 2023-11-21

**Authors:** Laura Parra-Anguita, María Dolores López-Franco, Juan Miguel Martínez-Galiano, Manuel González-Cabrera, Sara Moreno-Cámara, Nani Granero-Moya

**Affiliations:** 1 Department of Nursing, Faculty of Health Sciences, University of Jaén, 23071 Jaén, Spain; lparra@ujaen.es (L.P.-A.); jgaliano@ujaen.es (J.M.M.-G.); mgonzale@ujaen.es (M.G.-C.); smcamara@ujaen.es (S.M.-C.); jgranero@ujaen.es (N.G.-M.); 2 Consortium for Biomedical Research in Epidemiology and Public Health (CIBERESP), 28029 Madrid, Spain

**Keywords:** project-based learning, higher education, satisfaction, nursing, nursing homes

## Abstract

Project-based learning (PBL) is a teaching methodology that allows students to acquire knowledge and competencies through the completion of projects that respond to real-life problems. The aims of this study were to evaluate the acquisition of knowledge of students of the Aging Nursing subject through a PBL-based intervention and determine the degree of student satisfaction with the use of this methodology. A mixed, quasi-experimental, pre–post study was conducted without a control group using an educational intervention based on PBL and descriptive phenomenology with content analysis of the experiences reported after it. A knowledge questionnaire about nursing homes was administered before the start of the intervention. After using PBL to carry out the subject project, the same knowledge questionnaire and an ad hoc questionnaire on satisfaction, assessment, and improvement aspects were administered. In total, 111 nursing students participated. The difference in knowledge after the educational intervention was significant. The mean pre-intervention score was 5.56, SD 1.50, and the mean post-intervention score was 7.14, SD 1.59, (*p* = 0.001). In total, 74% of the students stated that they were very satisfied with the use of this methodology. The students had a positive perspective on the process of acquiring knowledge that PBL allows. The students improved their knowledge about the planning and management of nursing homes with the use of the project-based learning teaching methodology. They were very satisfied with said activity. Teachers must be adequately trained for the correct implementation of this teaching methodology. This study was not registered.

## 1. Introduction

Project-based learning (PBL) is a teaching methodology that allows students to acquire knowledge and competencies through the development of projects that respond to real-life problems. The PBL methodology is considered an innovation in higher education and facilitates the development of professional competencies in university students [1]. 

PBL can potentially increase students’ sense of responsibility and control over their learning. PBL provides an environment in which students are engaged in a continuous hands-on activity in which they give and receive constructive feedback, guidance, and support from other students and teachers. Multiple interactions are also performed in addition to conflict resolution [2]. Likewise, the exploratory process involved in PBL can develop multiple cognitive abilities and problem-solving abilities, thus achieving effective learning [3]. 

The use of PBL in mixed learning environments, where different teaching methodologies centered on the student or the teacher are combined, allows nursing students to acquire practical skills that provide many benefits for nursing work in real-care settings. PBL is associated with “students’ ability to discover problems”, while practical training based on experience is associated with “students’ ability to sustain action”. This didactic methodology focuses on the creation and development of specific projects that should not be confused with another similar methodology in terms of student participation and motivation, which is problem-based learning aimed at solving problems [4]. 

Research in nursing students through project-based learning is being used through different methodological designs. We found studies with a quasi-experimental study design using a pretest–posttest design of a non-equivalent control group [5] or accounts of experiences on the use of PBL in the course of educational actions in nursing practice [6]. PBL is also being used in professional-level training for quality-of-care improvement by using project-based learning to teach advanced practice nurses [7].

This methodology has been used in research at the university level with Nursing degree students [2,4], Early Childhood Education students [1,8], and Pedagogy degree students [9], as well as in different geographical areas, such as Japan [4], Taiwan [3], the United States [10], and Spain [8,9].

Project-based learning is perceived as more effective at increasing knowledge acquisition and problem-solving compared to other methodologies, such as conferences, flipped classrooms, or problem-based learning [10]. In addition, students are more satisfied with courses that use this student-centered learning pedagogy [10,11]. The effects of PBL are difficult to specify as they depend on the subject areas, the duration of the implementation in the classroom, and the circumstances of the institution or center where they are carried out [12], as well as the size of the group, the use of technological support, or the educational stage where it is implemented [13].

As a consequence of the aging population and social changes in family structures, many of the elderly are cared for in nursing homes. There is a great lack of knowledge about the functioning and organization of these institutions among nursing students. This aspect made us consider the organization of the Aging Nursing subject (this is the name given to Gerontological Nursing in our curriculum) to address issues such as the preparation and organization of nursing homes, their financing, and resources (professional and material) as well as the quality of care they should provide using this active learning methodology based on projects through the creation of a nursing home for the elderly. Therefore, the following objectives were proposed:

− Evaluate the acquisition of knowledge about nursing homes for older adults using the PBL methodology.
− To assess the experience and satisfaction with the use of the PBL teaching method in the Aging Nursing subject of the Nursing degree.

## 2. Materials and Methods

### 2.1. Design

This research used a mixed-methods approach combining a quantitative and a qualitative study. First, a quasi-experimental, pre–post study without a control group was developed through an educational intervention based on PBL with students of Nursing of Aging in the Nursing degree at the University of Jaén (Spain). This was followed by a descriptive phenomenological study of the experiences reported after the educational intervention.

### 2.2. Sample

The target population included all the students enrolled in the Aging Nursing course at the University of Jaén: a total of 133, of whom 106 were women and 27 were men, and their ages ranged from 20 to 52 years. This subject is taught in the third year of the nursing degree, which consists of 4 courses. It was decided to include all these students since the completion of the project was part of the overall evaluation of the course. The final sample consisted of students who voluntarily participated in the research.

### 2.3. Procedures

In the subject of Nursing Aging, topics related to physiological changes in the aging process, the most prevalent diseases in the elderly, or social and healthcare resources in the community are dealt with. In relation to this last aspect, the project is developed through the PBL methodology. The students were divided into 30 work groups. The students self-selected into groups of 4 to 5 students. The random selection of students was not possible; therefore, it is a non-probabilistic convenience sample. Each work group had to carry out a project that consisted of planning and managing a residential care facility for older adults where quality gerontological care would be provided. Every decision in the planning and management of the nursing home had to be based on the necessary gerontological care. This project was carried out during the course of the entire subject (one semester), and it was elaborated through PBL. The students received a document that provided general indications for the elaboration of the work. It included the structure to be presented, information on the requirements for the presentation, and the way in which the evaluation would be carried out. In order to ensure that all the members of the group participated equally, they were told that the final grade was individual. The calculation of the final grade was the result of the project grade (group work) and a personal grade (±2 points on the group grade) obtained from the evaluation of the presentation and a defense made by each of the students in the group. The project should have contained the following sections: Section 1.—Opinions or previous knowledge about what a nursing home for the elderly is; Section 2.—Research and information search; and Section 3.—Development of the project of the nursing home (organization, operation, staff, programs, activities, etc.). A maximum length of 15 pages was required for the Word document. The main researcher and coordinator of the subject in question carried out two mandatory group tutorials with all the students, where both the purpose of the project and the indications to carry it out were explained as well as the possibility of participating in the research on a voluntary basis. The realization of the project was mandatory as it was part of the evaluation of the subject. 

The 30 groups made the final presentation of the project, for which they had about 20 min to present their project and 10 min of debate and discussion with the rest of their classmates and teachers. On the first day of class, all the students filled out a self-administered multiple-choice questionnaire on knowledge related to nursing homes. At the end of the project (ten weeks later), and after their presentation, they all filled out the same knowledge questionnaire and a survey, which collected their degree of satisfaction, their perspectives on the most positive and negative aspects of carrying out the project, and improvements to be implemented. Both questionnaires were developed by the research team.

### 2.4. Instruments 

The data were collected through self-administered questionnaires prepared ad hoc through an online tool (Google form) for subsequent analysis. The knowledge questionnaire consisted of 11 multiple-choice questions with 4 response options where only one was true. The content of the questionnaire was based on the main topics of the subject and, in particular, on the aspects relevant and necessary for the development of the project. The maximum achievable score was 11 points. The second questionnaire addressed the satisfaction of the students using a 5-point Likert scale (from “not at all satisfied” to “very satisfied”) in relation to carrying out this training activity and their perception of the organization and planning of the information presented by the students themselves (from “very bad” to “very good” or “I would have liked the structure to be previously determined by the teacher”). Open questions were asked about the most positive and negative aspects of carrying out the training activity, the suggestions for the next PBL project, and the usefulness of the PBL methodology (Table 1).

### 2.5. Data Analysis

The data normality was verified using the Kolmogorov–Smirnov test. Both univariate and bivariate analyses were performed. Descriptive analysis of each variable was performed by calculating the measures of frequency and percentage for the qualitative variables and the measures of central tendency and dispersion for the quantitative variables. To compare the means of knowledge before and after the intervention, the Student’s t test or the Wilcoxon test was used, after checking for normality. Inadequately or incompletely completed questionnaires were excluded. The software package SPSS v.24 was used to perform the analyses. The significance level was set at 0.05.

The responses of the qualitative dimensions were analyzed by means of a content analysis based on the proposal of Taylor et al. [14]. The qualitative analysis of the speeches followed an iterative process in several stages: exhaustive reading of the texts, fragmentation of those considered significant into units of analysis, and subsequent coding. Finally, the codes were grouped into thematic categories taking the open questions asked in the questionnaire as a reference. To support the credibility of the study, triangulation was performed in the analysis by two different researchers [15]. 

### 2.6. Ethical Considerations

At the beginning of the project, the students were informed of the objectives, and their participation was voluntary at all times. By completing the questionnaires, they gave their consent to participate in the research. The students had the right to withdraw at any time without affecting their academic situation. The confidentiality of their personal data was maintained. The data collected was used for specific research purposes and is kept in the custody of the researchers. Ethical approval was granted by the Research Ethics Committee of the University of Jaen (SEPT.22/1.PRY).

## 3. Results

The final study sample consisted of 111 students enrolled in the Aging Nursing course (133 enrolled). The response rate was 83.4%.

Of these, 78.03% were women, while 21.97% were men. The age range was from 20 to 52 years. The mean age was 22.51 (SD 5.85).

### 3.1. Knowledge Acquisition

The mean pre-intervention score was 5.56 (SD 1.50), and the mean post-intervention score was 7.14 (SD 1.59). The distribution of the score difference variable had a different distribution than the normal KMO (0.095 *p* = 0.015), so the Wilcoxon non-parametric tests were performed, showing the existence of a significant difference between them (Z = −6.018 *p* = 0.001).

Regarding the questionnaire items, we can say that the item that showed the greatest learning was item 2 “*Which of the following services is mandatory for a residential care facility for older adults with more than 60 users*” (only 2 students answered it correctly in the pre-questionnaire and 54 in the post-questionnaire). The item that most students answered correctly in the pretest was item 5 “What is the primary objective of active aging?” (106 students answered it correctly in the pre-questionnaire and 111 in the post-questionnaire). The item with the least variation in terms of the number of correct answers was item 11 “The mistreatment suffered due to abandonment or failure to fulfill the care obligations of a person is called” (the number of correct answers only increased by two between the pre and post-questionnaires).

### 3.2. Student Satisfaction

Regarding the questionnaire on satisfaction with carrying out this learning activity, 74.4% indicated that they were *Very satisfied*, 24.8% were *Satisfied*, and 0.8% were *Somewhat satisfied*. When asked what they thought of deciding on the organization and planning of the information presented, 65.9% stated that it was *Very good*, 20.2% stated *Good*, 13.2% stated *I would have liked the structure to be previously determined by the teacher*, and 0.8% thought it was *Very bad*. Another aspect asked was their opinion about carrying out this group project. In total, 76% stated it was *Very useful*; 23.3% stated it was *Useful*, and 0.8% stated it was *Not at all useful*.

The open questions inquired about the students’ experience in carrying out the proposed activity using PBL. The categories and subcategories emerged as shown below.

#### 3.2.1. Positive Aspects/Strengths of Work Using PBL

The students had a positive perspective regarding the operation of nursing homes (protocols, organization, strengths, and weaknesses), the work carried out by different professionals within them, and the possibilities for improvement that they present or the real needs of older adults (Table 2). Carrying out the project contributed to modifying many students’ perceptions about the reality of nursing homes/residential care facilities for older adults. The knowledge acquired allowed them to identify which organizational and care models are the most appropriate to implement in a residential facility for older adults, as well as the elements for improvement.

The following are some examples of student comments:

(A positive aspect has been) “*knowing the current situation regarding the nursing homes and knowing how they are organized, care and activities offered*”.

“*Be aware that (nurses) must be the axis of care for older adults*”.

“*A positive aspect is the change in mentality regarding nursing homes*”.

“*Positively assess the function of a nursing home*”.

The students’ positive remarks about the project include an emphasis on the acquisition of the predetermined competencies, the teamwork that allows sharing and discussing different experiences and opinions, and the joint exposition that facilitated hearing the proposals and reflections of their classmates. The promotion of critical thinking, the autonomy to carry out the activity or the construction of one’s own learning, and the awareness of the evolution in their knowledge about the needs of older adults also emerged.

“*Sharing our ideas generates a broader and more open knowledge about nursing homes*”.

“*By getting involved, we learn so much more*”.

“*The freedom for creativity has allowed us to really enjoy it*”.

#### 3.2.2. Negative Aspects/Weaknesses of PBL Work Project

Regarding the weaknesses or negative aspects of the project, the students especially referred to the need to spend a lot of time carrying it out, as well as the little time allocated for presenting their project. In some cases, they alluded to the proposed page limit. For some, it was insufficient, while for others it was excessive. Also, they referred to a lack of specific instructions on the development of the project. 

“*The negative aspect is that it has taken me a considerable time of work*”.

“*We didn’t have the time to be able to present well what our ideal nursing home would be like*”.

“*I would have liked a slightly longer page limit*”.

On the other hand, they found that similar ideas appeared during the presentation of the different groups, and this repetition was considered a negative point. In addition, some of the common drawbacks of joint work were pointed out, such as the difficulty in meeting or coordinating tasks. 

“*It gets a bit repetitive because the ideas are similar*”.

“*It is difficult to meet to do group work*”.

Another issue the students reported was the frustration that appears when confronting their work with reality and verifying that it remains more on the plane of the ideal or utopian.

“*We have proposed an ideal nursing home, and in reality, they really are not like we suggest*”.

Finally, there were those who thought that the information provided to carry out the activity was a negative aspect, as they considered that it was insufficient and inaccurate to develop the project.

“*I didn’t have much information on how to do the project*”.

#### 3.2.3. Improvement Suggestions for Future Projects

The students considered that the following proposals could be interesting for future editions of the activity:

Regarding the development of the activity and to enrich the final work:

Many students considered it important to incorporate the opinions of different actors: relatives of the older adults or the workers. They stated that they would especially like to know what older people think. For this, they proposed a visit to a nursing home or requested guest speakers to explain their experiences. A conversation or interview with older adults could offer an important basis to help build the project from their preferences, desires, or real needs.

“*Bring older people to talk about how they live in a nursing home*”.

“*Approach from different perspectives: students, parents, grandparents...*”

Likewise, they also mentioned that in order to have more points of view and reduce repetition, some students could carry out a negative residential care facility project, that is, pointing out those aspects that should never exist in a center. Along these lines, they suggested inverting the approach: by first identifying the negative aspects present in some residential care centers and proposing improvements from there. They also indicated that it would be interesting if specific aspects on which to focus the design of the nursing home were distributed among the different work groups. This would allow for deeper knowledge and better variation in the projects.

“*Create a nursing home to which we would not like to go*”.

“*That each group do a different directed project, that is, an ideal residence for older adults, another depending on the workers, another how the families see it*”.

In addition, they proposed carrying out research on residential facilities in other environments and on different centers, such as day centers. Delving further into the nursing care that is provided there was also suggested.

“*Propose that different residential facilities in different countries be investigated*”.

“*Delve deeper into the work of the residential care nurses*”.

In relation to the project, its structure, or programming:

The students suggested that the time devoted to carrying out the project should be greater, as well as the duration of its presentation. With respect to the length, linked to one of the negative aspects, some believed that the pages of the report should be limited while there are those who believed that, on the contrary, they should be expanded.

“*Provide more time for projects*”.

“*Expand the extension and exposure time*”.

Finally, and repeatedly, many students proposed greater exhaustiveness in the information offered to carry out the work: more instructions, more specificity, greater clarification of the content, carrying out a group tutorial before beginning, or even a guide on how to perform it. 

“*Explain something else in the informative document and not only the sections that we have to follow*”.

“*Explain more the contents that must be included*”.

“*Put a clearer guide for what needs to be done*”.

“*Put in the instructions what general sections we must include in the work*”.

As one of the students explained, “We are used to being given very detailed guidelines, and the moment they leave us a little more to our devices, we get lost.”

## 4. Discussion

After carrying out the intervention, a significant increase in the level of knowledge on the subject studied was observed. Therefore, project-based learning is ideal for increasing knowledge about essential aspects of institutions dedicated to caring for older adults. 

Although the difference in knowledge after the educational intervention was statistically significant, the authors expected that the final mean knowledge would have been higher and, thus, that the students would have experienced significant learning. The students did not have previous experience in the use of this didactic methodology, so it is believed that the results were not influenced by this aspect. We recommend further research comparing different teaching methodologies in order to be able to demonstrate which one produces greater learning.

In Garnjost’s study, which compared four different student-centered methodologies (problem-based, service-learning, flipped classroom, and project-based) with lectures (teacher-centered pedagogy), the students only perceived that project-based learning had a significant impact on problem-solving and knowledge acquisition compared to lectures and that the satisfaction was significantly higher than the other methodologies [10].

Research that has used this same teaching methodology but online affirms that the use of PBL promotes the development of teamwork skills and encourages autonomy, proactivity, commitment, respect for the opinion of others, and the exercise of creativity [6], coinciding with the findings of our research.

In the use of the PBL methodology, the supervisory role of the tutor becomes essential, involving students in the creation and execution of the project [6]; the tutor must be trained to effectively guide the teamwork of undergraduate nursing students throughout the PBL process so that they achieve their goal [16]. This aspect makes the use of this methodology difficult because it requires greater involvement of the tutor and adequate training to establish a connection with the real world and promote the transfer of knowledge to practical situations, as well as greater involvement of the students.

The majority (74.4%) stated they were very satisfied with this training activity. There are also other studies along the same lines, in which PBL has been used in the university setting. These studies conclude that PBL produces an increase in the motivation and commitment of the students and has a positive effect on the knowledge of fundamental content and the development of the so-called 21st-century skills, such as collaboration, critical thinking, autonomy, problem-solving, time planning, or the ability to express oneself adequately [1,3,9,17]. 

Although PBL is a difficult methodology to apply in higher education, the results show the advantages that this type of methodology provides and the high level of satisfaction of the participating students [1] (79.6%) [8]. When exploring the association between group work skills and satisfaction with PBL in the university setting, the results show that there is a significant and positive relationship between both [18].

Regarding the qualitative study of the experience in the nursing home project, the open questions allowed us to explore the students’ experiences. On the one hand, the question about the positive and negative aspects of carrying out the activity gives us a subjective assessment of the task carried out, which is something that we consider important to reveal the perception that emerges from the speeches. On the other hand, the question about suggestions for improvement for future activities places us in the scenario of reflection on the work and the projection of ideas acquired both in the preparation of the project itself and in the presentation of the different groups. Some suggestions for improvement made by the students require commenting on ethical considerations, such as conducting a “negative” nursing home project or making visits to nursing homes to analyze them. If these proposals were to be carried out, it would have to be made clear that when bad examples of care were detected, they would be acted upon.

One of the aspects valued as positive has to do with group work. Collaborative learning work is dynamic, and the need for students to work together to achieve common goals favors the development of knowledge and skills and generates a profitable and positive interdependence for students [18]. 

In addition, our study, like that of Mora [19], found that the student feels satisfied with this methodology as they become an active subject within their training, and they appreciate the development of skills such as critical thinking. This methodology improved the critical thinking and autonomy of the students by encouraging their active participation and motivation to learn. Also, a greater emotional involvement was generated, since the students were involved in the creation and execution of a project that was relevant and interesting to them about the needs of older adults.

On the other hand, the use of this didactic methodology results in the achievement of different specific learning results regarding the subject of Aging Nursing, such as describing the structures and operation of the centers where care is provided to older adults. And, in achieving certain competencies, such as demonstrating the ability to gather and interpret the relevant data to make judgments that include a reflection on social, scientific, or ethical issues. In addition to this, other training is carried out in generic or transversal skills, such as resource management, public speaking skills, decision-making, or interpersonal relationships.

Although, in general, PBL turns out to be a positive experience in terms of the time allotted for carrying out the activity, it can lead to it being perceived as unsatisfactory, and, in this regard, our results coincide with what was stated by Hernando et al. [20].

Proposing a change in methodology, encouraging active learning [5], and offering the freedom to interpret the required content can generate some difficulty and restlessness in those who are used to greater precision. In addition, that which, in principle, we consider as an opportunity to expand the possibilities of intervention is considered a burden that generates insecurity and requires greater involvement of both teachers and students. 

Among the limitations is the small size of the sample. The possibility of generalizing the results is limited because the study population was limited to the students of a single subject of the Nursing degree at the University of Jaen. Finally, it is important to consider the limitations of using ad hoc questionnaires as well as the use of a self-reported survey method as a data collection system for both quantitative variables and qualitative dimensions.

## 5. Conclusions

The students of the Aging Nursing subject of the Nursing degree at the University of Jaen improved their knowledge about the planning and management of nursing homes with the use of the project-based learning teaching methodology. The students who participated in the project were very satisfied with the activity. The students had a positive perspective on the process of acquiring knowledge with PBL, with in-depth learning of some aspects, such as the operation of nursing homes, the work carried out by different professionals within them, and the needs of older adults as well as the possibilities of the improvement of these institutions. In terms of the learning methodology, they considered group work and the active role of students to be positive. They assessed the need to spend a lot of time to carry it out or the lack of precise indications about the development of the work as negative aspects.

It is recommended that faculties should facilitate teacher training in this teaching methodology in order to be able to implement similar projects with guaranteed success.

More research is needed to compare project-based learning with other teaching methodologies in order to determine which one is more recommendable.

## Figures and Tables

**Table 1:** Questions from both questionnaires.

Pre–Post Questionnaire Elderly Nursing Homes
1. The minimum ratio of nurses required in a nursing home in a situation of dependency is: 2. Which of the following services is mandatory for a nursing home with more than 60 residents? 3. The following principles shall govern the operation of nursing homes for the elderly in a situation of dependency: 4. Which of the following protocols should be in the Junta de Andalucía’s nursing homes? 5. What is the primary goal of active aging? 6. The “giants of geriatrics” are: 7. Regarding Comprehensive Geriatric Assessment, point out the WRONG statement. 8. How often should an ear canal examination be recommended for the elderly population? 9. Which of the following technical aids does the Junta de Andalucía provide subsidy for? 10. Which of the following is not an objective of preventive gerontology? 11. Abuse suffered due to abandonment or neglect of duties in the care of a person is referred to as:
**Satisfaction Questionnaire. Nursing Homes Project**
Indicate the degree of satisfaction with this training activity. Indicate the most positive and negative aspects of this training activity (indicate at least 1 positive and 1 negative aspect). What suggestions for improvement do you have for future calls? How did you feel about deciding on the organization and planning of the information presented? Doing this project as a group has seemed to me: Working with this project-based learning methodology has helped you to... (you can check more than one option)

**Table 2:** Positive aspects of work using PBL.

Related to Residential Care Facilities	
Knowledge Regarding	Functioning Professionals’ work Needs of older adults Improvement possibilities in nursing homes
Recognizing The Value of Nursing Homes	
Project as a Facilitator of a Change in Perspective	
**Regarding the Learning Method**	
Group Work	Shared experiences Distribution of work
Active Role of Student	Development of own learning Awareness of learning evolution Autonomy Satisfaction Encouragement of critical thinking

## Data Availability

The datasets used and analyzed during the current study are available from the corresponding author on request.

## Public Involvement Statement

There was no public involvement in any aspect of this research.

## Guidelines and Standards Statement

This manuscript was drafted against the Strengthening the Reporting of Observational Studies in Epidemiology (STROBE) for the quantitative study and Consolidated Criteria for Reporting Qualitative Research (COREQ) for the qualitative study.
